# Supplementary material for: CALMODULIN1 and WRKY53 Function in Plant Defense by Negatively Regulating the Jasmonic Acid Biosynthesis Pathway in Arabidopsis
Source: Int J Mol Sci. 2022 Jul 13;23(14):7718. doi: 10.3390/ijms23147718 (PMC9323616; doi:10.3390/ijms23147718)
Supplement: Supplementary file 1 [file ijms-23-07718-s001.zip › ijms-1719670-supplementary.pdf]

Supplement

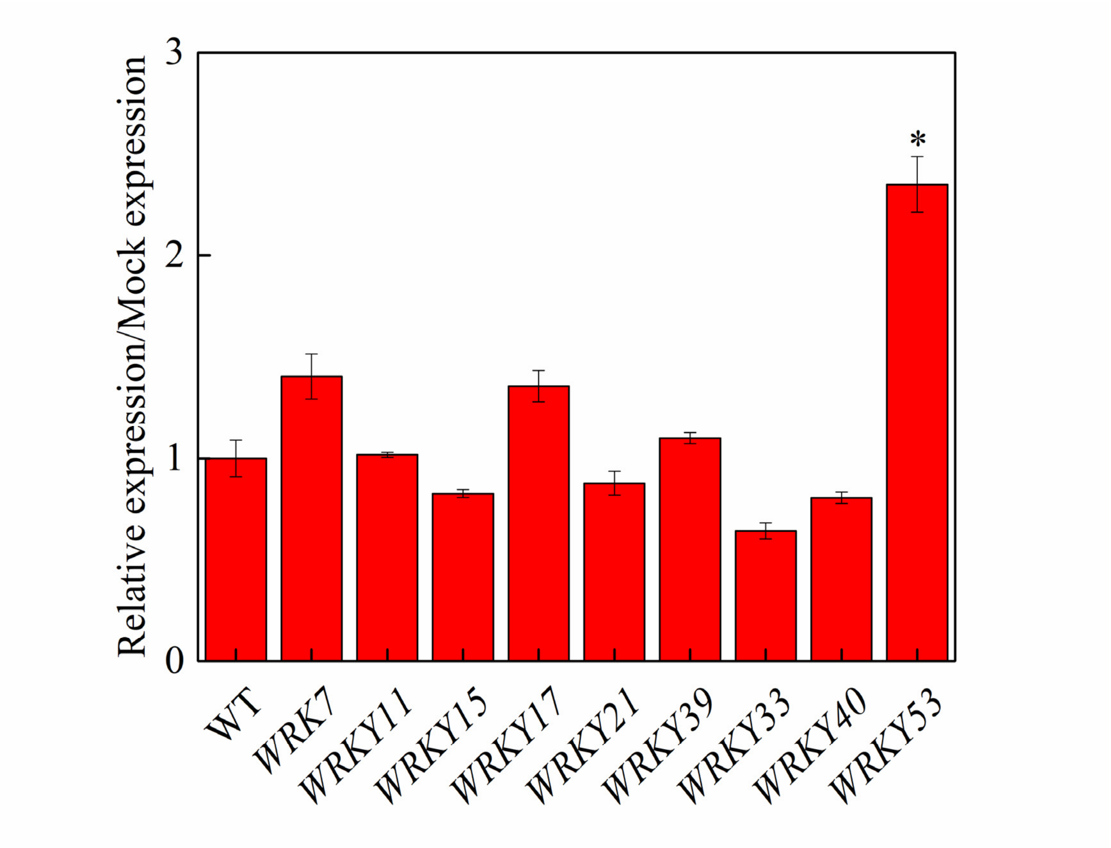

Figure S1. Selection of *WRKY53* based on the results of gene expression analysis in *cam1* mutant compared with WT. Each group had three biological replicates and every replicate had more than 30 plants. Plants were grown for 2 weeks. Error bars denote ± SEM, * is significantly increased at $p < 0.05$, Dunnett's C (variance not neat).

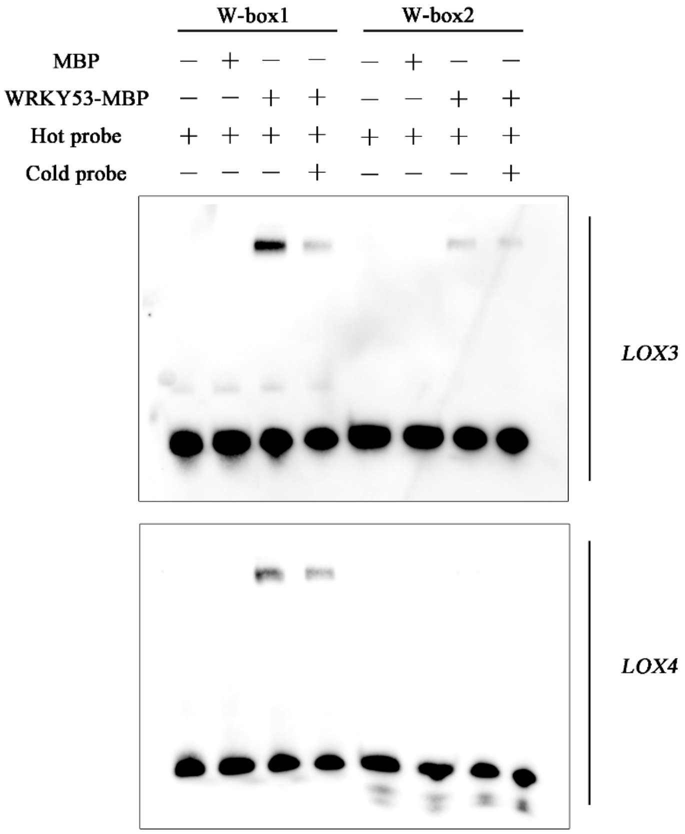

Figure S2. EMSA shows that WRKY53 binds to the W-boxes in the *LOX3* and *LOX4* promoters. every promoter has two W-boxes, W-box 1 and W-box 2.

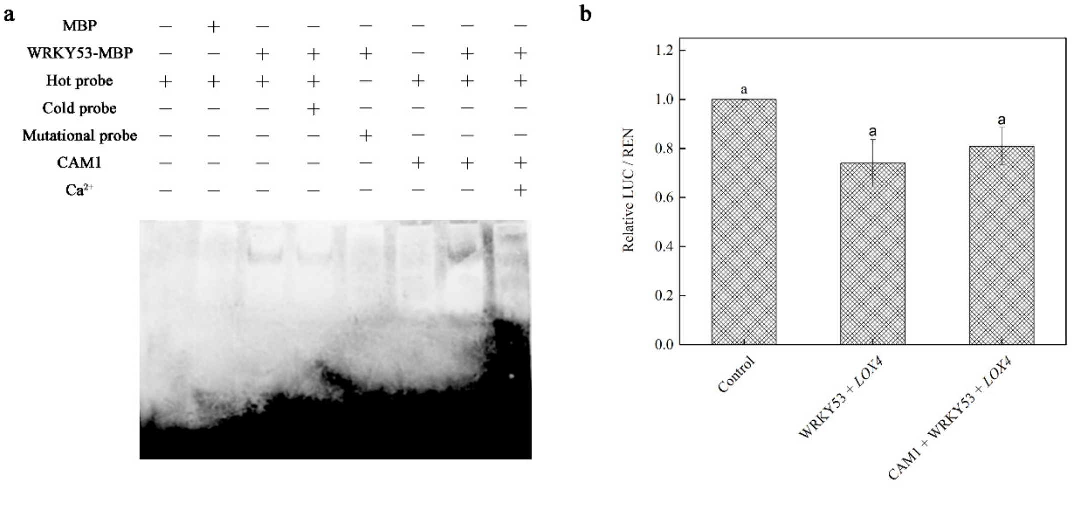

Figure S3. Interaction between WRKY53 and the *LOX4* promoter. (a) EMSA assay

shows that WRKY53 has a weak interaction with the *LOX4* promoter. And it shows the effects of calcium and CAM1 on the binding between WRKY53 and the first W-box in the *LOX4* promoter. A hot probe refers to a biotin-labeled probe and a cold probe to an unlabeled probe (200-fold the concentration of hot probe). Ca^{2+} concentration is 10^{-2} mM. (b) Luciferase activity assay shows that WRKY53 interacts with the *LOX4* promoter, WRKY53 negatively affects *LOX4* expression, and CAM1 does not significantly decrease the negative regulation. Error bars denote ± SEM, columns labeled with different letters are significantly different at $p < 0.05$, Dunnett's C (variance not neat).

Table S1. The probe sequences in the EMSA.

W-boxes	Probe sequence (5'-3')
LOX3 W-box1-F	CACACTTATATTTTGACCCTATGTTACT
LOX3 W-box1-R	AGTAACATAGGGTCAAAATATAAGTGTG
LOX3 W-box2-F	CGTAACTCCGTTTGACTCAATTACCCCA
LOX3 W-box2-R	TGGGGTAATTGAGTCAAACGGAGTTACG
LOX4 W-box1-F	TTTGTATATGTTTGACTTTGTGGCTATT
LOX4 W-box1-R	AATAGCCACAAAGTCAAACATATACAAA
LOX4 W-box2-F	CGGAACTCAGTTTGACTCAAACTAGAAG
LOX4 W-box2-R	CTTCTAGTTTGAGTCAAACTGAGTTCCG
